# Conversion From Intravenous In-Hospital Belatacept Injection to Subcutaneous Abatacept Injection in Kidney Transplant Recipients During the First COVID-19 Stay-at-Home Order in France

**DOI:** 10.3389/ti.2023.11328

**Published:** 2023-07-24

**Authors:** Dominique Bertrand, Mélanie Brunel, Ludivine Lebourg, Anne Scemla, Mathilde Lemoine, Lucile Amrouche, Charlotte Laurent, Christophe Legendre, Dominique Guerrot, Dany Anglicheau, Rebecca Sberro-Soussan

**Affiliations:** ^1^ Department of Nephrology, Kidney Transplantation and Hemodialysis, Rouen University Hospital, Rouen, France; ^2^ Department of Kidney Transplantation, Hôpital Necker-Enfants Malades, Assistance Publique-Hôpitaux de Paris, Université Paris Cité, Paris, France; ^3^ INSERM U1096, University of Rouen Normandy, Rouen, France

**Keywords:** COVID-19, kidney transplantation, belatacept, abatacept, CNI toxicity

## Abstract

The first COVID-19 stay-at-home order came into effect in France on 17 March 2020. Immunocompromised patients were asked to isolate themselves, and outpatient clinic visits were dramatically reduced. In order to avoid visits to the hospital by belatacept-treated kidney transplant recipients (KTRs) during the initial period of the pandemic, we promptly converted 176 KTRs at two French transplant centers from once-monthly 5 mg/kg in-hospital belatacept infusion to once-weekly 125 mg subcutaneous abatacept injection. At the end of follow-up (3 months), 171 (97.16%) KTRs survived with a functioning graft, 2 (1.14%) had died, and 3 (1.70%) had experienced graft loss. Two patients (1.1%) experienced acute T cell–mediated rejection. Nineteen patients (10.80%) discontinued abatacept; 47% of the KTRs found the use of abatacept less restrictive than belatacept, and 38% would have preferred to continue abatacept. Mean eGFR remained stable compared to baseline. Seven patients (3.9%) had COVID-19; among these, two developed severe symptoms but survived. Only one patient had a *de novo* DSA. Side effects of abatacept injection were uncommon and non-severe. Our study reports for the first time in a large cohort that once-weekly injection of abatacept appears to be feasible and safe in KTRs previously treated with belatacept.

## Graphical Abstract

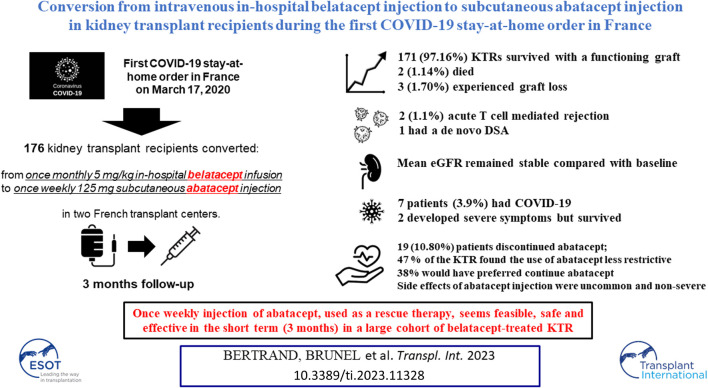

## Introduction

Belatacept is a fusion protein composed of the heavy chain constant region of human IgG1 linked to the extracellular domain of human cytotoxic T lymphocyte-associated antigen 4 (CTLA-4) that selectively inhibits T-cell activation through costimulation blockade. Since its approval by the U.S. Food and Drug Administration and the European Medicines Agency in 2011, belatacept has become widely used in kidney transplantation as an alternative to calcineurin inhibitors (CNIs) for maintenance immunosuppression [1, 2]. Belatacept is used as a *de novo* immunosuppressive therapy after kidney transplantation, but also as a conversion from calcineurin inhibitors [1, 2] in cases of CNI toxicity and/or side effects [3]. Belatacept should be administered intravenously every month under the supervision of a healthcare provider.

Coronavirus disease 2019 (COVID-19) has been particularly deleterious in kidney transplant recipients (KTRs), with a very high risk of severe disease associated with a high mortality rate [4, 5]. During the first wave of the pandemic, a lockdown order came into effect in France on 17 March 2020. Immunocompromised patients were asked to isolate themselves and outpatient clinic visits were dramatically reduced. Patients who have converted to belatacept for CNI toxicity are suspected to be at high risk of opportunistic infections [6, 7]. In order to avoid frequent clinic visits by belatacept-treated KTRs and prevent SARS-CoV-2 contamination during the initial pandemic period, and also to release some institutional resources to care for COVID-19-infected KTRs, we searched for a temporary alternative solution to monthly administration of belatacept. CNI conversion appeared to be a safer option because i) patients could take their treatment orally at home; ii) most of them had previously received tacrolimus or cyclosporine before belatacept conversion; and iii) CNIs significantly reduce acute rejection rates. However, this solution was not generalizable to all of our patients, due to some having a low estimated glomerular filtration rate (eGFR) or history of CNI toxicity and/or intolerance [8].

Abatacept is also genetically constructed by fusion of the external domain of human CTLA-4 to the heavy chain constant region of human IgG1. This drug was the predecessor of its higher-affinity evolution, belatacept, which was engineered to contain two amino acid substitutions to bind its ligands CD80 and CD86 with greater potency for use in kidney transplantation. Abatacept was approved by the Food and Drug Administration for use in adults with rheumatoid arthritis in 2005 [9] and in children with juvenile idiopathic arthritis in 2008 [10], and it can be used intravenously or subcutaneously [11]. Data on the use of abatacept after kidney transplantation are very scarce, but the results of a preclinical study using a primate kidney transplant model [12] and of a small report on nine patients [13] seemed reassuring.

Considering these results, and replicating the protocol used for rheumatoid arthritis [11], we believed that once-weekly subcutaneous injection of abatacept could be a safe, effective, and logistically feasible alternative to belatacept during the COVID-19 pandemic. Here, we report on a cohort of patients from two transplant centers who received abatacept during the initial stay-at-home order in France. Our aim was to assess short-term graft and patient outcomes, kidney allograft function, immunological features, and tolerance and safety of abatacept maintenance to provide a rationale for belatacept avoidance in the event of a prolonged crisis, and as an alternative in KTRs with problematic vascular access; these findings have even greater relevance in the current period of belatacept shortage.

## Material and Methods

### Patients

**FIGURE 1:**
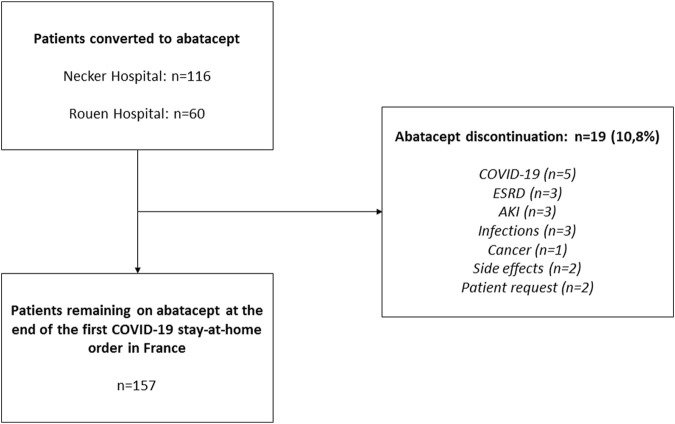
Flow Chart. AKI, acute kidney injury; eGFR, estimated glomerular filtration rate; ESRD, end-stage renal disease; SLKT, simultaneous liver–kidney transplant.

A total of 176 KTRs receiving belatacept as a conversion protocol at two French transplant centers (Necker University Hospital and Rouen University Hospital) were converted to abatacept during the COVID-19 pandemic (Figure 1). All patients were 18 or older, had received either a living or a deceased donor kidney transplant, and had received no prior or concurrent non-renal solid organ transplant. Patient characteristics and biological data were collected from electronic medical records. According to French law (loi Jardé), because this was an anonymous retrospective study, institutional review board approval was not required.

### Immunosuppression

Patients had been initially converted from CNI to belatacept according to the protocol published in phase II and III conversion studies. Belatacept was then maintained at 5 mg/kg every 4 weeks in all KTRs. For abatacept conversion, patients received subcutaneous injection of 125 mg abatacept once weekly at home, initiated 1 month after the last belatacept infusion. The remaining components of maintenance immunosuppression were not modified while the patients were on abatacept.

### Follow-Up

Patients were followed up 3 months after the first injection while on abatacept therapy. After 3 months, patients were switched back to belatacept because the French administration authorized the in-home infusion of belatacept in the context of the COVID-19 pandemic. Kidney allograft function was assessed on day 0 and at 3 months, using plasma creatinine and the Chronic Kidney Disease Epidemiology Collaboration (CKD-EPI) equation [14] for eGFR in KTRs with a functioning graft. BK virus and cytomegalovirus (CMV) viral loads were measured. The tolerance and safety of abatacept maintenance were evaluated using a specific questionnaire.

### Anti-HLA Antibody Testing

The presence of anti–HLA-A, -B, -Cw, -DR, -DQ, and -DP donor-specific antibodies (DSAs) was analyzed using single-antigen flow bead assays (One Lambda, Inc., Canoga Park, CA) performed using a Luminex platform on serum samples at time of transplantation, every year or at the time of any biopsy, and 3 months after abatacept conversion. The presence of DSAs was defined by a median fluorescence intensity (MFI) ≥500. The number, class, specificities, and MFI of each anti-HLA DSA were recorded.

### Histologic Phenotyping of Kidney Allograft Biopsies

During the 3 months under abatacept, graft biopsies were performed only for cause. Biopsies were graded using the 2017 Banff classification [15]: C4d staining was performed by immunohistochemistry on paraffin sections or immunofluorescence on frozen sections and graded from 0 to 3 by the percentage of peritubular capillaries with linear staining.

### Statistical Analysis

Continuous variables were summarized in the form of means with SDs or medians with IQRs, and they were compared using Mann-Whitney or t-tests, as appropriate. Categorical variables were summarized in the form of numbers with proportions, and they were compared using Fisher’s exact test. We used STATA (version 14, Data Analysis and Statistical Software) and R (version 3.2.1; R Foundation for Statistical Computing) to carry out descriptive analyses. A *p*-value <0.05 was considered significant.

## Results

### Baseline Characteristics of Converted KTRs

A total of 176 patients from two transplant centers were converted to abatacept during the early stages of the COVID-19 pandemic in France (March 2020). Of these, 19 patients (10.80%) discontinued abatacept: 12/116 patients (10.34%) at Necker Hospital and 7/60 (11.67%) at Rouen Hospital (*p* = 0.8). Detailed reasons for abatacept discontinuation are presented in Figure 1. The remaining 157 patients (89.20%) were reassessed 3 months after conversion. KTR characteristics are listed in Table 1.

**TABLE 1:** Transplant recipients’ demographic and baseline characteristics.

	All patients *n* = 176	Necker *n* = 116	Rouen *n* = 60	*p*-value
Recipient				
Age, yr, median (IQR)	57 (44–66)	54.5 (43–65)	59.5 (47–68)	0.096
Men, *n* (%)	111 (63.07)	70 (60.34)	41 (68.33)	0.326
ESKD causes, *n* (%)				
Diabetes/hypertension	38 (21.59)	18 (15.52)	20 (33.33)	0.092
Glomerulonephritis	37 (21.02)	22 (18.97)	15 (25.00)	
Interstitial nephritis	19 (10.80)	15 (12.93)	4 (6.67)	
Polycystic kidney disease	27 (15.34)	19 (16.38)	8 (13.33)	
Uropathy	13 (7.39)	11 (9.48)	2 (3.33)	
Other	8 (4.55)	6 (5.17)	2 (3.33)	
Unknown	34 (19.32)	25 (21.55)	9 (15.00)	
Previous kidney transplant, *n* (%)	20 (11.36)	16 (13.79)	4 (6.67)	0.212
Donor				
Age, yr, median (IQR)	62 (51–71)	61.5 (50–71.5)	62 (52–70.5)	0.815
Men, *n* (%)	86 (48.86)	59 (50.86)	27 (45.00)	0.526
Deceased donor, *n* (%)	153 (86.93)	95 (81.90)	58 (96.67)	*0.005*
Preformed anti-HLA DSAs^a^, *n* (%)				
Class I		6 (5.26)		
Class II		13 (11.40)		
Class I/II		2 (1.75)		
Induction treatment^a^, *n* (%)				
Thymoglobulin®	62 (35.43)	43 (37.39)	19 (31.67)	0.285
Basiliximab	109 (62.29)	68 (59.13)	41 (68.33)	
None	4 (2.29)	4 (3.48)	0 (0.00)	

DSA, donor-specific antibody; ESKD, end-stage kidney disease; HLA, human leukocyte antigen.

^a^ Missing data: preformed DSAs, two patients; induction treatment, one patient.

Italic values indicate statistically significant between the two groups.

Conversion data are outlined in Table 2. Belatacept indications were similar between centers, but belatacept conversion occurred later in Rouen. All except two patients (2.5 and 4 months) were converted to abatacept beyond the first 6 months after transplant.

**TABLE 2:** Conversion data.

	All patients *n* = 176	Necker *n* = 116	Rouen *n* = 60	*p*-value
Time of belatacept conversion post-Tx, mo, median (IQR)	17 (5–57)	13.1 (3–44)	30 (9–104)	*0.001*
Belatacept indication, *n* (%)				
CAD – IFTA	139 (78.98)	86 (74.14)	53 (88.33)	0.133
CNI toxicity	24 (13.64)	18 (15.52)	6 (10.00)	
TMA	10 (5.68)	9 (7.76)	1 (1.67)	
*De novo*	3 (1.70)	3 (2.59)	0 (0.00)	
Time of abatacept conversion post-Tx, mo, median (IQR)	60 (32–95)	55 (32–85)	66 (32–123)	0.184
Time of abatacept conversion post-belatacept, mo, median (IQR)	25 (11–48)	30 (15–51)	19 (6–42)	0.*008*
Immunosuppression regimen, *n* (%)				
Abatacept/Mycophenolic acid/Prednisone	108 (61.36)	88 (75.86)	20 (33.33)	*< 0.001*
Abatacept/Azathioprine/Prednisone	14 (7.95)	13 (11.21)	1 (1.67)	
Abatacept/Everolimus/Prednisone	4 (2.27)	3 (2.59)	1 (1.67)	
Abatacept/Mycophenolic acid	26 (14.77)	1 (0.86)	25 (41.67)	
Abatacept/Azathioprine	2 (1.14)	0 (0.00)	2 (3.33)	
Abatacept/Everolimus	1 (0.57)	0 (0.00)	1 (1.67)	
Abatacept/Prednisone	20 (11.36)	11 (9.48)	9 (15.00)	
Abatacept	1 (0.57)	0 (0.00)	1 (1.67)	
Maintenance drug doses, median (IQR)				
Mycophenolic acid, mg/d	720 (450–720)	720 (540–1,080)	720 (360–720)	0.077
Azathioprine, mg/d	100 (75–125)	100 (75–150)	100 (50–100)	0.576
Everolimus trough level, ng/mL	5.1 (5–7.1)	5 (3–7.1)	6.25 (5.1–7.4)	0.248
Prednisone (mg/d)	7.5 (5–10)	10 (5–10)	7.5 (5–10)	*0.021*
Time on abatacept, mo, median (IQR)		2.9 (2.8–3.7)		
Number of abatacept infusions, median (IQR)		12 (11–16)		

CAD, chronic allograft dysfunction; CNI, calcineurin inhibitor; IFTA, interstitial fibrosis and tubular atrophy; TMA, thrombotic microangiopathy; Tx, transplant.

Italic values indicate statistically significant between the two groups.

### Patient and Graft Outcomes After Conversion

At the end of follow-up, 171 patients (97.16%) survived with a functioning graft, 2 (1.14%) died, and 3 (1.70%) experienced graft loss. Causes of death were vascular (stroke) and infectious (invasive aspergillosis with CMV disease). Graft loss only occurred in patients with chronic allograft dysfunction and severe renal impairment at baseline (eGFR <20 mL/min/1.73 m^2^). These patients returned to hemodialysis and underwent premature discontinuation of abatacept. No biopsy was performed.

Eight patients (4.55%) developed an acute kidney injury requiring a graft biopsy under abatacept. Detailed histologic findings are presented in Table 3. Two patients experienced acute T cell–mediated rejection (TCMR). The first of these patients experienced a grade Ib TCMR (Biopsy#4) 2 months after abatacept conversion and was successfully treated with a high dose of intravenous steroids. Abatacept was stopped and belatacept was resumed. The second experienced a grade IIb TCMR (Biopsy#7) 1.5 months after abatacept initiation; this was successfully treated with steroids. Belatacept was also resumed, but the patient developed severe invasive aspergillosis with CMV disease and died. A third patient was diagnosed with chronic antibody-mediated rejection (Biopsy#4). Abatacept was pursued and eGFR remained stable.

**TABLE 3:** Banff classification of kidney graft biopsies performed under abatacept treatment.

Biopsy#	t	i	g	ah	v	ti	iIFTA	cg	ci	ct	cv	mm	cpt	C4d
1	0	0	0	2	0	0	0	0	0	0	2	0	0	0
2	0	0	1	2	0	1	1	0	2	2	1	1	0	0
3	0	0	1	3	0	0	1	3	2	2	3	1	2	0
4	3	2	0	2	0	2	3	0	1	1	1	0	1	0
5	1	0	0	2	0	0	1	0	2	2	1	0	0	0
6	0	0	0	2	0	0	2	0	1	1	2	0	0	0
7	3	1	0	1	2	2	3	0	2	2	2	1	0	0
8	0	0	0	3	0	1	2	0	3	3	2	0	0	0

ah, arteriolar hyaline thickening; cg, transplant glomerulopathy; ci, interstitial fibrosis; cpt, peritubular capillary inflammation; ct, tubular atrophy; cv, arterial fibrous intimal thickening; g, glomerulitis; i, interstitial inflammation; t, tubulitis; v, endarteritis.

**FIGURE 2:**
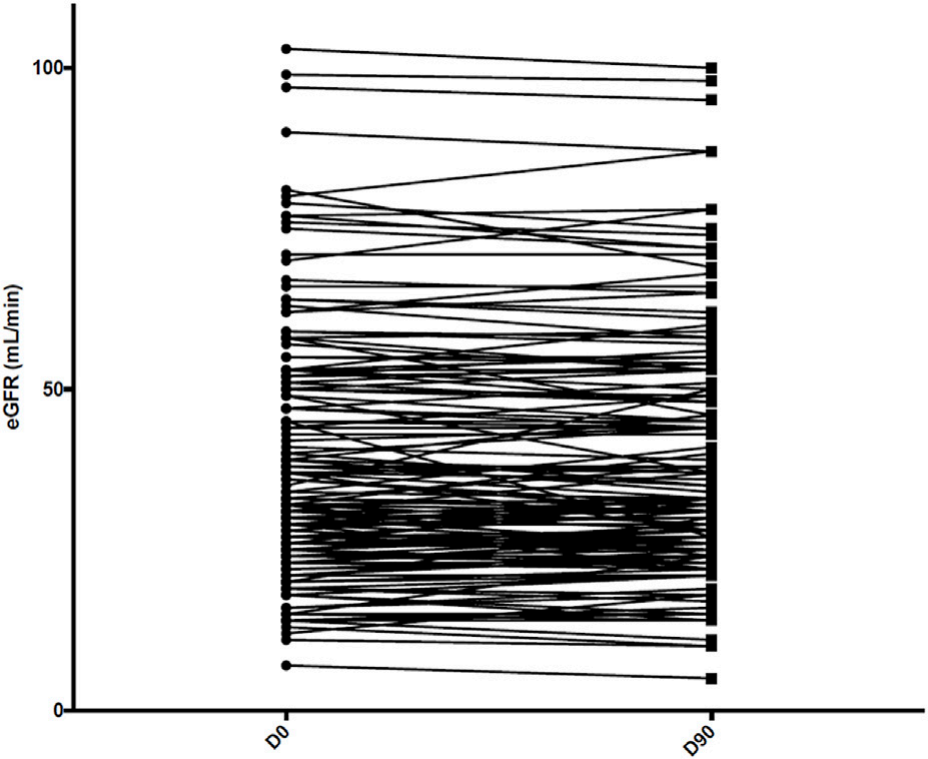
Kidney allograft function assessed at day 0 (D0) and at 3 months (D90) among the 157 patients receiving abatacept at the end of follow-up. eGFR: estimated glomerular filtration rate.

Among the 157 patients receiving abatacept at the end of follow-up, mean eGFR remained stable compared with baseline (38.0 ± 18.9 mL/min/1.73 m^2^ versus 38.1 ± 19.4 mL/min/1.73 m^2^, *p* = 0.8) (Figure 2), as did proteinuria/creatininuria ratio (0.56 ± 0.65 g/g versus 0.58 ± 0.85 g/g, *p* = 0.6). Only one patient had a *de novo* DSA, without a history of antibody-mediated rejection.

### Tolerance and Safety of Abatacept

One patient (0.57%) experienced CMV disease of the gastrointestinal tract under abatacept, which resolved with a single course of ganciclovir therapy for 3 weeks and MPA discontinuation; additionally, three patients (1.71%) contracted an asymptomatic CMV viremia (>3 log copies/mL), of whom two had already had CMV viremia under belatacept. One patient had a low-level BK viremia without nephritis. Seven patients (3.98%) experienced COVID-19 under abatacept; among these, two developed severe and critical symptoms but survived. Five KTRs developed other non-severe viral infections: one simplex herpes virus and one zoster herpes virus infection; one adenovirus cystitis; one norovirus colitis; and one gastroenteritis. Bacterial infections occurred in 14 KTRs: 10 non-severe urinary tract infections, one bacteremia, one pneumonia, one *clostridium difficile* colitis, and one *campylobacter* colitis. Finally, fungal infections occurred in two patients: one case of invasive aspergillosis and one extensive dermatophytosis.

**FIGURE 3:**
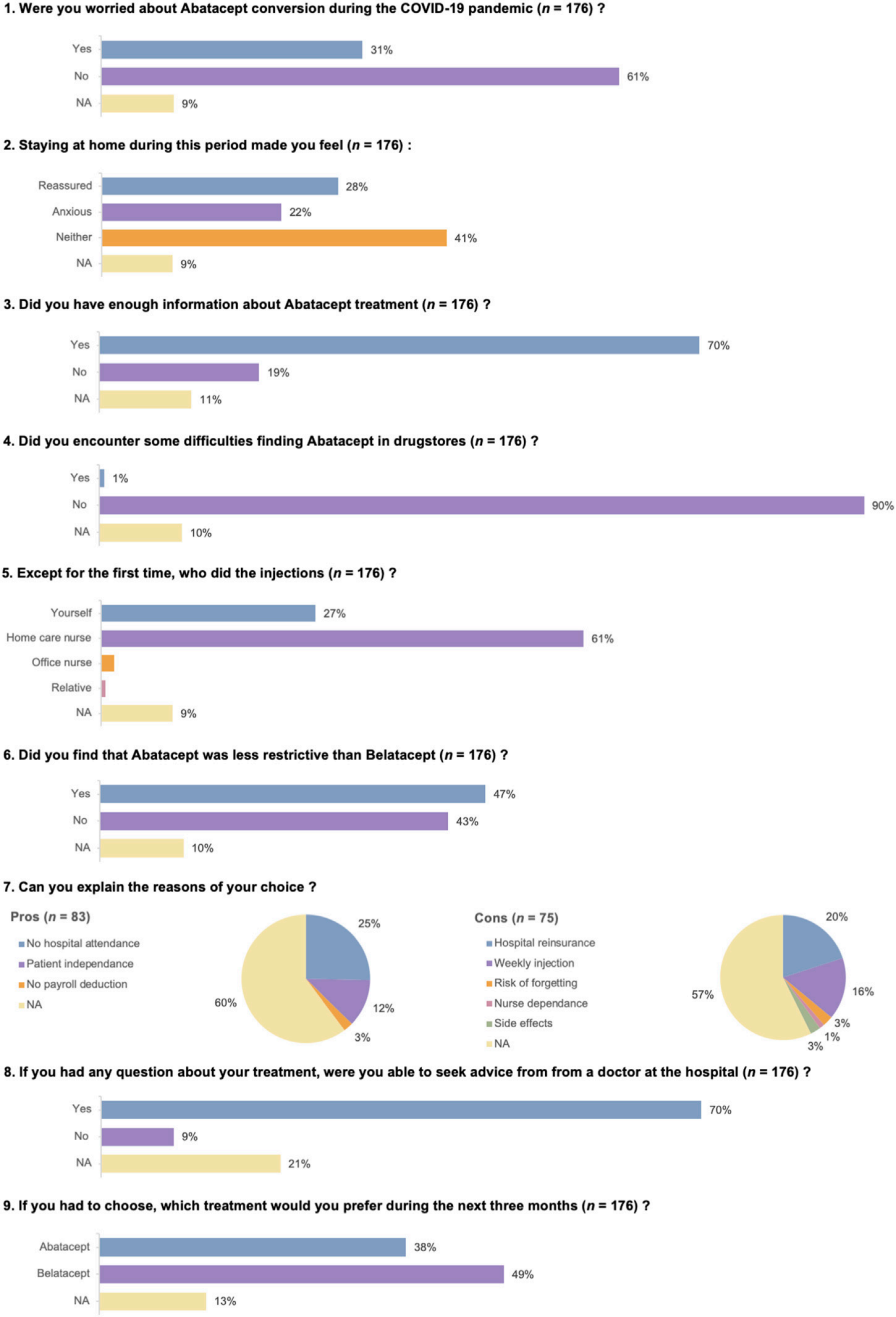
Quality of life survey.

Side effects of abatacept injection were uncommon and non-severe. These are reported in Table 4. The results of the quality of life survey are depicted in Figure 3.

**TABLE 4:** Side effects under abatacept treatment.

	Yes	No	Missing
Infusion site reaction, *n* (%)	7 (3.98)	150 (85.23)	19 (10.80)
Arthralgia, *n* (%)	25 (14.20)	131 (74.43)	20 (11.36)
Erythema, *n* (%)	7 (3.98)	151 (85.80)	18 (10.23)
Abdominal pain, *n* (%)	16 (9.09)	144 (81.82)	16 (9.09)
Diarrhea, *n* (%)	20 (11.36)	140 (79.55)	16 (9.09)
Nausea/vomiting, *n* (%)	10 (5.68)	149 (84.66)	17 (9.66)
Stomatitis, *n* (%)	6 (3.41)	152 (86.36)	18 (10.23)
Cough, *n* (%)	11 (6.25)	148 (84.09)	17 (9.66)
Headache, *n* (%)	19 (10.80)	139 (78.98)	18 (10.23)

## Discussion

To the best of our knowledge, we report here for the first time in a large cohort of KTRs the feasibility, safety, and efficacy of conversion from once-monthly intravenous infusion of belatacept to once-weekly subcutaneous injection of abatacept as a maintenance immunosuppression regimen. Tolerance was excellent and side effects were very uncommon in this fragile population. eGFR remained stable during the follow-up period, and cases of biopsy-proven TCMR after abatacept conversion were rare (1.1%). In comparison, conversion from belatacept to another immunosuppressive treatment in cases of CNI toxicity or intolerance is associated with a decrease in eGFR, as recently reported by [8]. In this cohort of 44 KTRs from five French transplantation centers, who were converted from maintenance belatacept to another regimen because of a complication (n = 28), by patient request, or due to belatacept shortage (n = 13), mean eGFR decreased from 44.2 ± 16 mL/min per 1.73 m^2^ at conversion from belatacept to 35.7 ± 18.4 mL/min per 1.73 m^2^ at last follow-up (*p* = 0.0002). Of note, eGFR decreased more severely in patients who were converted to CNIs.

As an alternative approach, we could have increased the spacing of the belatacept injections from 4 to 8 weeks, as reported by [16]; however, although the result was not statistically significant, rates of BPAR were twofold higher in patients administered belatacept every 8 weeks vs. every 4 weeks. Another alternative would have been to pursue in-hospital belatacept infusions with a specific infection control protocol, as reported by Kamar et al. [17]. Nevertheless, these measures were very restrictive and time-consuming, and they did not fully rule out the risk of nosocomial transmission of SARS-CoV-2. Our in-home attitude is also retrospectively supported by the low humoral and cellular immunogenicity induced by SARS-CoV-2 vaccination in belatacept-treated KTRs [18, 19], related to their profoundly immunocompromised condition and to their high risk of opportunistic infection [6, 7] and severe COVID-19.

Data on the use of abatacept after kidney transplantation are very scarce. Abatacept is a genetically constructed by fusion of the external domain of human CTLA-4 to the heavy chain constant region of human IgG1. This drug was the predecessor of its higher-affinity variant belatacept, which was engineered to contain two amino acid substitutions to bind its ligands CD80 and CD86 with greater potency for use in kidney transplantation. Apprehension toward its use after kidney transplantation is therefore related to its supposedly insufficient immunosuppressive capacity. The data reported in the present study are quite reassuring, with a low risk of rejection when abatacept is used in a conversion protocol beyond the first 6 months after kidney transplant. Although preclinical non-human primate (NHP) studies have shown superior results with belatacept in a kidney transplant model [20], abatacept has also exhibited efficacy in a kidney transplant model [12], as well as in an NHP allogeneic islet transplant model, as a *de novo* monotherapy or in combination with CD154‐ specific blockade [21]. It has also since been effectively used in the clinical setting to treat rheumatoid arthritis and, more recently, other autoimmune disorders [9]. While belatacept may be indeed more potent and the preclinical data on abatacept warrant caution regarding its immunosuppressive strength for the purpose of inhibiting alloreactivity, preclinical data from murine and primate models alike have proven not to be entirely predictive of clinical outcomes or directly translatable to humans [22]. Abatacept has been used after kidney transplantation for recurrence of focal and segmental glomerulosclerosis [23], but most publications are case reports [24, 25] and its effectiveness is widely debated [26, 27]. Recently; [13], have reported on a series of 9 CNI‐intolerant transplant recipients who were converted to abatacept early after transplant as a form of rescue immunosuppression during periods of belatacept shortage. A retrospective review revealed successful allograft salvage and 100% patient and graft survival (median 115 months) after conversion to abatacept. Patients received intravenous abatacept for a median duration of 82 months with stable, long‐term renal allograft function, a single cellular rejection episode, and no clinically apparent protective immunity concerns. Furthermore, CD86 receptor saturation levels (a pharmacodynamic measure of costimulation blockade proposed to correlate with inhibition of alloresponses [28]) did not differ between belatacept‐ and abatacept-treated patients tested after infusion. Although abatacept was originally formulated as an infusion, it is now available in a subcutaneous formulation, which has equal safety and efficiency in rheumatoid arthritis patients [11]. Nevertheless, only one patient was treated with once-weekly subcutaneous injections of abatacept, although this patient was treated under this regimen for 16 months without complications. Very recently, Uro-Coste et al. reported their experience with abatacept injection in 5 KTRs, suggesting that weekly subcutaneous administration of 125 mg abatacept may be an effective alternative to belatacept [29]. The data presented here on the use of subcutaneous injection of abatacept in a large cohort could be very useful for patients with CNI toxicity or intolerance and in need of conversion to belatacept but with poor vascular access, like many end-stage renal disease patients. This approach could also represent a solution in cases of belatacept shortage. Costimulation blockade with abatacept could potentially have a logistical advantage over belatacept in kidney transplant recipients. Nevertheless, our niche experience over 3 months does not allow us to make definitive assertions as to the potential benefits mentioned above.

Our work has several limitations. The short duration of our follow-up period (3 months) prevents us from drawing a firm conclusion on the risks of rejection and infection in patients treated with abatacept as a maintenance therapy. Nevertheless, the median half-life of belatacept is reported to be 8 days (range: 3.1–11.9) [30], and the very low incidence of TCMR during the weeks following abatacept initiation can be taken into account and is quite reassuring. Because of the ethical issues related to data scarcity on abatacept safety, and the possibility of in-home belatacept infusion, we could not accept the risk of continuing to pursue abatacept therapy once the first wave of SARS-CoV-2 had ended. The absence of a control group receiving ongoing belatacept injection is also problematic. However, our main goal was to avoid frequent hospital visits by immunocompromised KTRs during the initial period of the SARS-CoV-2 pandemic. Under these conditions, we chose to treat as many patients as possible with abatacept. A clinical trial NCT04955366 (https://clinicaltrials.gov/) was developed to answer the question of whether patients can be safely converted from monthly belatacept IV infusions to subcutaneous abatacept injections without a decrease in kidney function. The results of this study will be available in late 2024 or in 2025. In the meantime, the message of our work is not to treat all belatacept-converted patients with subcutaneous abatacept, as reflected by the questionnaire completed by the patients: 49% preferred in-hospital belatacept, and 43% did not find that abatacept was less restrictive than belatacept. Nevertheless, we would like to point out that before the COVID-19 pandemic, all patients on belatacept were receiving infusions in hospital. As a result of these treatment sessions, they were closely monitored on a monthly basis. The pandemic has taken patients away from the hospital, making them very anxious at times and most probably explaining the rather low acceptance rates and the rather high discontinuation rate over this short period. In this context of in-home subcutaneous abatacept injection, close monitoring with, for example, regular blood draws or regular teleconsultation could be reassuring for patients and represent an additional safety measure.

In conclusion, our study demonstrates for the first time, in a large cohort of belatacept-treated KTRs, that once-weekly injection of abatacept, used as a rescue therapy, appears to be feasible, safe, and effective in the short term (3 months). The current context of belatacept shortage makes this report even more important.

## Data Availability

The original contributions presented in the study are included in the article/supplementary material, further inquiries can be directed to the corresponding author.

## Abbreviations

CNI, calcineurin inhibitor; DSA, donor-specific antibody; eGFR, estimated glomerular filtration rate; KTR, kidney transplant recipient; COVID-19, coronavirus disease 2019; CTLA-4, cytotoxic T lymphocyte-associated antigen 4; CMV, cytomegalovirus; TCMR, T cell–mediated rejection.
